# Cardiac ECM: Its Epigenetic Regulation and Role in Heart Development and Repair

**DOI:** 10.3390/ijms21228610

**Published:** 2020-11-15

**Authors:** Rui Song, Lubo Zhang

**Affiliations:** Department of Basic Sciences, Lawrence D. Longo, MD Center for Perinatal Biology, School of Medicine, Loma Linda University, Loma Linda, CA 92350, USA

**Keywords:** extracellular matrix, cardiac development, regeneration, remodeling, epigenetics

## Abstract

The extracellular matrix (ECM) is the non-cellular component in the cardiac microenvironment, and serves essential structural and regulatory roles in establishing and maintaining tissue architecture and cellular function. The patterns of molecular and biochemical ECM alterations in developing and adult hearts depend on the underlying injury type. In addition to exploring how the ECM regulates heart structure and function in heart development and repair, this review conducts an inclusive discussion of recent developments in the role, function, and epigenetic guidelines of the ECM. Moreover, it contributes to the development of new therapeutics for cardiovascular disease.

## 1. Introduction

The extracellular matrix (ECM) is made up of many proteins that hold together and direct cell adhesion and migration, as well as regulate cellular growth, metabolism and differentiation signals, and cell functions, in healthy and pathological conditions [1,2,3,4,5,6,7]. Cells that lose contact with the ECM via integrins have a higher chance of undergoing apoptosis (programmed cell death) than anchored cells. Cell adhesions intervene in effective bidirectional communications among cells and the extracellular network. ECM–cell interaction and ECM-mediated cell–cell communication play crucial roles in modulating cell adhesion, motility, survival, proliferation, differentiation, and maturation [8,9]. Utilizing integrins and non-integrin receptors (e.g., dystroglycan, sulfatides discoidin domain receptors, CD44, epidermal growth factor receptor, and P-selectin glycoprotein ligand-1) [10,11], cells can detect the physical and biochemical properties of the extracellular framework. The ECM is a highly dynamic structure present in all tissues, and maintains the structure and function of the organ, mediating the development and remodeling of the organ.

**Figure 1:**
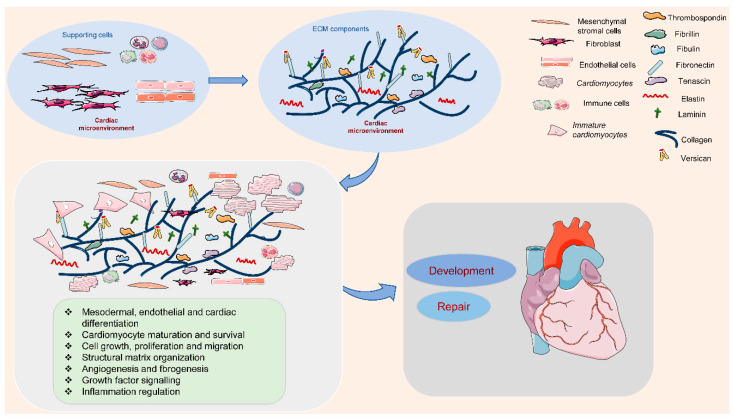
Extracellular matrix (ECM) components and their role in the cardiac microenvironment during heart development and repair. In the cardiac microenvironment, supporting cells, including mesenchymal stromal cells, fibroblasts, endothelial cells, and immune cells, produce the main ECM proteins. These ECM components promote cardiomyocyte differentiation, maturation, and survival, and the interaction between cardiomyocytes and supporting cells, contributing to heart development and repair.

The ECM is outlined because of the cell-free elements secreted by cells that consist of macromolecules like scleroprotein, collagens, proteoglycan, hyaluronan, non-collagenous glycoproteins, and proteinases [12,13]. In the cardiac microenvironment, non-myocyte cell types populate the cardiac interstitium [14]. The heart surface is covered by epicardium, a derivative of mesothelial cells. It is termed proepicardium, for its function in giving rise to epicardium and epicardium-derived cells [15]. The cells migrate to the myocardial wall and differentiate into fibroblasts, endothelial cells, and smooth muscle cells [15]. These cells produce and release most matrix proteins, and the cell–ECM communication has an essential role in the programming and development of heart function (Figure 1). Among them, fibroblasts are the major cell type contributing to the ECM synthesis, in order to maintain the myocardial tissue architecture and mediate cell signaling through growth factor interactions and integrins [16]. Human mesenchymal stromal cells can release ECM proteins such as fibronectin (FN) and collagens into the space around cells to promote cell spreading [17]. Endothelial cells are also crucial in vascularization, cardiac function, and/or remodeling by producing ECM proteins such as collagens, laminin, elastin, fibulins, proteoglycans, matrix metalloproteinases (MMPs), tenascin-C (TNC), and thrombospodins (TSPs) [18]. In addition, immune cells in the cardiac microenvironment can also produce ECM proteins such as MMPs to modulate the immune response in the heart, contributing to the regulation of cardiomyocyte survival [19]. Therefore, cell-derived ECM and related signaling play an essential role in regulating cardiovascular function from early development to postpartum life, aging, and possibly disease.

In this review, we will focus on the role of the ECM in the regulation of cardiac development and repair. In addition, we will also discuss the underlying mechanisms of epigenetic regulation of ECM in the heart, and the potential clinical implications of ECM based therapeutic approaches for cardiovascular disease.

## 2. The Role of ECM in Heart Development

The ECM provides essential organic components for embryogenesis and tissue maturation. The ECM is conditional; the slightest changes in its physiological state result in ruinous consequences, which might lead to severe defects or even death of the developing embryo (Table 1). In the middle development, the mechanism of dorsal closure could also be a sophisticated method, involving associate degree orchestration of cell–matrix interaction between smooth muscle cells, epithelial tissue cells, and the ECM [20]. A recent study demonstrated that nucleus–cytoskeleton–ECM connections triggered coordinated cardioblast movements, and controlled cardioblast number in Drosophila [21].

Inherent cardiovascular disease is the leading non-infectious rationalization for death in children. It is becoming apparent that many internal organ abnormalities once thought to possess complex etiologies occur because of mutations in biological process management genes [66]. These mutations are manifested at birth as grievous internal organ malformations, or later as subtler internal organ abnormalities. Understanding the role of ECM in internal organ development has vital implications not only for an understanding inherent upset, but also for the chance of internal organ repair through genetic reprogramming of non-cardiac cells to a cardiogenic role strategic location.

The ECM gene expression profiles of embryonic and adult mouse cardiac fibroblasts revealed that higher levels of *FN1*, collagen genes, *TNC*, *Postn* (periostin), and *Hapln1* (hyaluronan and proteoglycan link protein 1) were expressed in embryonic than adult hearts [67]. Importantly, embryonic cardiac fibroblasts promote cardiomyocyte proliferation through fibronectin and collagen, involving β1 integrin signaling, leading to myocardial growth and ventricular compaction during cardiogenesis [67]. In an environment rich in abnormal cells and growth factors, activated fibroblasts can produce matrix proteins, proteases and their inhibitors, and regulate matrix metabolism. Due to the pathological maturity, “stress shielding” of fibroblasts through the cross-linked matrix, and macromolecule withdrawal, may lead to quiescence and eventually apoptosis.

Fibulin belongs to a family of five extracellular glycoproteins and mediates the formation of proteoglycan aggregates, elastic fibers, fibronectin microfibrils, basement membrane networks, and supramolecular structures. The expression patterns of biological processes indicate that many fibrins are expressed at epithelial-mesenchymal transition sites during the entire embryogenesis, and the vascular system is related to one of these transition sites [68]. Fibulins 1 and 2 are highly expressed during cardiac valvuloseptal formation. Fibulin 1 is expressed by primordial vascular smooth muscle cells associated with the ventral endothelium of dorsal aortae and developing aortic-arch vessels [52]. In addition, fibulin 2 is expressed by coronary endothelial cells that originate from epicardial cells [69]. Interestingly, fibulin-1 deficiency, but not fibulin 2 deficiency, induced a perinatally lethal phenotype with a defective endothelial basement membrane of small vessels in mice [70]; this may be due to the functional compensation of fibulin 1.

Few studies have investigated the role of TSPs in cardiac development. Increased expression of TSP-1 in the second trimester was demonstrated to cause defects in the cardiovascular system and even embryonic lethality [71]. Conversely, the lack of TSP-4 led to increased ECM production and developmental heart enlargement [65]. As such, it is important to study the modular structures and binding interactions, and the temporal, spatial, and quantitative expression differences of various ECM proteins and their collaborations in cardiovascular health and diseases.

## 3. ECM in the Programming of Cardiovascular Repair

The adult heart has limited recovery and repair potential, and the loss of myocardial cells due to injury may end in heart disease and death. The cellular biological progression and restraining mechanisms associated with heart development and advancement can repair damaged adult hearts through the “stiring” pathway, which can determine the bioactivity during the entire embryogenesis. Incitement of the differentiation and proliferation of cardiomyocytes, by initiating the mitotic signaling pathway engaged with embryonic heart growth, points to a correlative methodology for heart recovery and repair [72]. Cardiac damage includes arterial sclerosis, myocardial infarction (MI), and ischemic and non-ischemic heart injury, which induces repair by the embryonic cell. Cells reply to the ECM by transforming their microenvironment, which becomes dysregulated in tube-shaped structural diseases, such as high blood pressure, restenosis, and arterial sclerosis [73].

**Figure 2:**
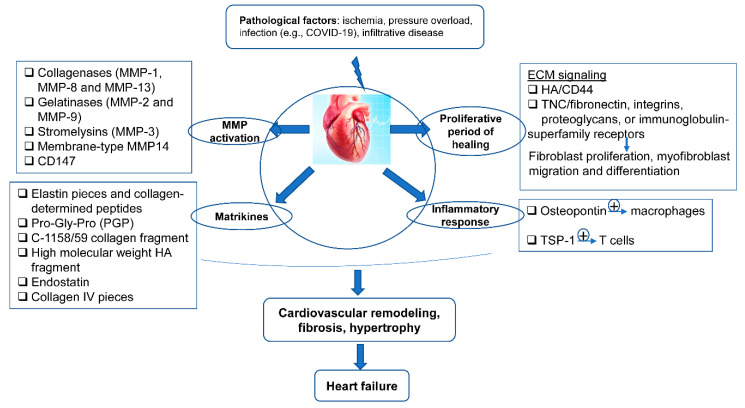
The ECM in the programming of cardiovascular repair and disease. Cardiovascular pathological factors induce abnormal synthesis and release of ECM proteins and ECM signaling, implicated in the process of matrix metalloproteinase (MMP) activation, matrikines production, proliferation, and inflammatory response; this results in cardiovascular remodeling, fibrosis, hypertrophy, and thus heart failure.

After MI, the ECM dynamic alteration and remodeling propels inflammation and repair [74,75]. The first generation of bioactive matrix fragments activates an unhealthy signal. An extremely plastic tentative matrix formation facilitates blood corpuscle infiltration and activates infarction myofibroblasts [76]. The deposition of matrix cellular macromolecules modulates growth factor signal transduction, and promotes the spatial and temporal regulation of the repair [77]. Temporal scales vary from conformational changes in control of the particle channel gap, to fibrillation over seconds, and end in death. Spatial scales vary from metric linear unit pore sizes in membrane channels and gap junctions, to the meter length scale of the whole cardiovascular system throughout a living patient. Overwhelming changes in the ECM composition are conducive to the pathologic process of cardiac remodeling (Figure 2).

### 3.1. Matrix Metalloproteinases Activation

Myocardial ischemia causes quick enactment of matrix metalloproteinases (MMPs), and the ensuing aging of framework pieces. MMP actuation has been identified in the heart interstitium as precisely as ten minutes after coronary impediment, preceding any proof of irreversible cardiomyocyte injury, and might be driven by ischemia-interceded ROS emissions [78,79,80]. Many MMPs correlate repeatedly with cardiomyocytes, endothelial cells, fibroblasts, as well as inflammatory leukocytes that penetrate the ischemic myocardium. Up-regulated collagenases (MMP-1, MMP-8, and MMP-13), gelatinases (MMP-2 and MMP-9), stromelysins (MMP-3), and membrane-type MMP14 have been found in the infarcted and post-MI remodeling myocardium [81,82,83,84,85,86]. Importantly, MMPs play an essential role in myocardial infarction. MMPs induce the production of cytokines, chemokines, and developmental factors through proteolysis, or destroy glycosaminoglycan restriction sites, interfering with the progress of chemokines fixing on the surface of endothelial cells, and enhance chemokines binding to leukocytes to control inflammation. In addition, MMPs can corrupt structural proteins, such as myosin, titin, and α-actinin, in cardiomyocytes. The grid autonomous activity of MMPs is generally of great significance in ischemic injury, and redesign after infarction remains obscure.

CD147 is a highly glycosylated transmembrane protein of the immunoglobulin superfamily, and the main MMP inducer. It is up-regulated in acute coronary syndrome and heart failure, and regulates MMP expression and ECM remodeling [87,88,89]. The up-regulation of CD147 and MMPs is closely related to inflammatory processes in cancer development [90,91]. Resident lung progenitor cells/stem cells that differentiate into myofibroblasts lead to lung fibrosis, a complication of coronavirus disease 2019 patients (COVID-19). The conceivable direct and indirect viral invasion of progenitor/stem cells through CD147 or ACE2 could give rise to reducing stem cell reserves, and hastening lung repair and regeneration [92]. It may thus be reasonable to speculate that COVID-19/CD147 may modulate MMP activity in cardiac injury and remodeling, and CD147/MMP could be a potential target for the treatment of COVID-19-related cardiovascular diseases.

### 3.2. Matrikines Lifespan

In harmed and renovating tissues, protease-interceded discontinuity of ECM proteins brings about the age of matrikines. Matrikines-specific receptors are involved in the process of ECM renewal, cellular proliferation, cellular migration, chemotaxis, and mitogenesis in association with inflammation, immune responses, organ development, wound repair, angiogenesis, atherosclerosis, tumor progression, and metastasis [93,94,95,96,97,98]. Elastin pieces and collagen-determined peptides are the best-studied matrikines, and are embroiled in the enactment of resistant cells and fibroblasts [98]. The matrikine acetylated Pro–Gly–Pro (PGP) induced vascular endothelial cell production of endothelin-1 by activating endothelial CXC chemokine receptor 2, leading to vascular inflammation and myocardial injury [99]. PGP requires the actuation of a multi-step course that includes prolyl endopeptidase, MMP8, and MMP9 to degrade collagens [100]. The proteolysis of laminins by MMP2 and MMP14 demonstrated robust neutrophil chemoattractant characteristics [101].

Even though the fast actuation of MMPs in the infarct is related to grid discontinuity, the role of these sections as bioactive pro-inflammatory matrikines has not been characterized. C-1158/59 collagen fragment was highly generated at day 7 post-MI. Significantly, exogenous delivery of p1158/59 peptide, mimicking the collagen fragment, could promote angiogenesis, and inhibit left ventricular (LV) remodeling and LV dysfunction [102]. In the ischemic myocardial microenvironment, a high molecular weight hyaluronic acid (HA) fragment induced expression of chemokines CCL2 (C-C motif chemokine ligand 2) and CXCL5 (C-X-C motif chemokine ligand 5) to promote M2 type macrophage polarization and neutrophil removal; this contributes to the suppression of the chronic inflammatory response and improves myocardial remodeling and myocardial function reconstruction [103]. Conversely, endostatin, a 20-kDa part of collagen XVIII, applies vigorous angiostatic activities and animates fibroblast expansion [104]. MMP9-intervened cleavage of collagen IV also creates pieces with angiostatic properties, such as tumstatin [105]. Matrikines may likewise balance fibroblast and vascular cell phenotypes. Endogenous matrikines’ job in the guiding of fibrogenic and angiogenic reactions following myocardial localized necrosis remains ineffectively comprehended. It will be interesting to determine whether pro-angiogenic matrikines can inhibit tissue necrosis induced by insufficient angiogenesis and chronic inflammation.

### 3.3. Regulation of Inflammatory Response

As mentioned above, matrikines-specific ECM protein fragments can enhance the inflammatory response by activating the innate immune response and mediating regulation of inflammatory cell apoptosis [106]. During pregnancy, immune cells penetrate and stay within the heart muscle, and function by modulating cardiac innate immune response throughout the entire life. Under cardiovascular pathologic conditions (e.g., myocardial infarct, infection, and infiltrative cardiac disease), many immune cells can be recruited to the myocardium to eliminate dying tissue, scavenge pathogens, and promote healing. Under some pathological conditions such as COVID-19 infection, immune cells cause irreversible harm, a tributary to heart failure [107].

Macrophages throughout repair express a secreted conjugated protein called osteopontin. It is concerned with cell adhesion and migration. There is a high expression of osteopontin template RNA and macromolecule in macrophages during the death of connective tissue throughout MI. The osteopontin is downregulated dramatically as a healing payoff, despite the macrophages. In vitro, fibrinogen animates cytokine emission by macrophages via TLR4 actuation [108]. During cardiac ischemia-reperfusion injury, fibrin D-dimers increased in plasm [109]. Inhibition of fibrin fragments with the peptide Bβ15–42T diminished infarct size and lessened leukocyte penetration through VE-cadherin in the heart, which was further confirmed in fibrin knockout mice [109]. However, the impacts of the peptide in a clinical preliminary trial were substantially less significant. Peptide organization in patients with ST-rise MI did not influence the infarct size through attractive reverberation imaging, nor decreased serum troponin I level [110].

End-stage non-ischemic heart failure patients have increased LV fibrosis, directly associated with T cell infiltration [111]. Recent studies clarified that T cells, especially T-helper cells and regulatory T cells, are essential regulators of the inflammatory and reparative responses, by providing signals for macrophages or fibroblasts [112,113,114], and improve cardiac regeneration after MI [112,115,116]. Either adoptive regulatory T cells transfer or the superagonistic antibody against CD28, a co-stimulator for T-cell activation and survival, diminished fibrosis and pro-inflammatory cytokine production, and improved cardiac function [117]. Thrombospondin-1, an essential matricellular protein, binding to CD47, induced T cell apoptosis and reduced T cell activation, resulting in limiting inflammation [106]. Recently, a potential new strategy engaged a membrane glycoprotein fibroblast activation protein (FAP), targeted to chimeric antigen receptor (CAR) T cells, and engineered to precisely ablate activated fibroblasts (myofibroblasts), which reduced the fibrotic burden in cardiac injury [118]. Due to the critical role of ECM and T cells in cardiac remodeling, the mechanisms underlying ECM–T cell interaction-mediated cardiac protection await necessary further elucidation.

### 3.4. ECM in the Proliferative Period of Healing

Dynamic changes in the ECM structure may add to reparative cell reactions during the proliferative period of a cardiovascular fix. Freeing of matrix parts by phagocytes may enact mitigating signals, smothering the enlistment of pro-inflammatory leukocytes. The lysis of the plasma-determined temporary framework, and conferred a grid arrangement, including cell proteoglycans, hyaluronan, fibronectin, and broad scope matricellular macromolecules that transduce development factor signs to reparative cells [119]. The dynamic regulation of ECM in the proliferation stage provides essential signals for converting fibroblasts into myofibroblasts. It may activate the angiogenesis pathways necessary for the development of new blood vessels along these pathways, thereby providing dynamic metabolic damage with oxygen and supplements.

Cardiac fibroblasts are major cellular effectors of internal organ repair; interactions with ECM proteins modulate their makeup and performance. They provide structural support for the attachment of internal organ cells throughout the development process, and specific growth factors and cytokines regulate the proliferation of embryonic cardiomyocytes. In postpartum life, internal organ fibroblasts play a vital role in the injury response [120]. The up-regulation of stromal cell macromolecules promotes signal transduction mediated by proteins and cytokines; this is due to the development of scars, lattice cross-connections, reduced stromal cell protein clearance, and reduced macromolecular signals, caused by deactivation and cell elimination of reparative localized necrosis fibroblasts.

Fibrosis is characterized by the accumulation of albumin and excessive ECM parts. This process has been compared to abnormal wound healing and abnormal heart remodeling/function. The main stage of wound healing involves ECM protein formation. Fibroblasts reside in the matrix and proliferate following the activation of leukocytes, which migrate to the wound and are maintained by the ECM. This corresponds with the presence of myofibroblasts, which are specialized and rationally formed cells. ECM signals and mechanical tension principally stimulate myofibroblast differentiation [121]. Clinical studies demonstrated that excessive myocardial collagen cross-linking was associated with myocardial fibrosis, and increased hospitalization risk for heart failure patients with hypertensive heart disease [122,123,124]. Collagens, the main ECM structural protein in the adult heart, and their signaling play an essential role in the fibroblast differentiation and proliferation during cardiac repair and remodeling. Type I and III collagens were demonstrated to affect fibroblast proliferation, while type VI collagen potently induced myofibroblast differentiation [125]. Another study showed that collagen I enhanced the differentiation and proliferation of myofibroblasts, through lowering α2β1 integrin expression and subsequently suppressing protein phosphatase type 2A activity and increasing protein kinase B activity [126]. Interestingly, a more recent study demonstrated that the pro-fibrotic factor, angiotensin II, induced collagen receptor cross-talk between discoidin domain receptor 2 and integrin-β1 in cardiac fibroblasts, leading to increased collagen I production and myocardial fibrosis [127]. ECM glycosaminoglycan HA and its receptor CD44 are involved in myofibroblastic activation [128]. TNC plays a control role in regulating embryonic development, wound repair, and regeneration, and tumor progression and metastasis. In cardiac development, TNC can provoke the initial differentiation of cardiomyocytes or coronary artery/angiogenesis. Although TNC is not expressed in healthy adult myocardium, it has been demonstrated that myocardial injury stimulates TNC expression. Consequently, TNC modulates the attachment of cardiomyocytes to connective tissue, augments myofibroblast migration and differentiation, and increases matrix metalloproteinases production, leading to tissue remodeling and healing [129,130,131].

In every cellular and extracellular event of physiology and pathology, ECM is the active player in ECM–ECM communication, cell–ECM communication, and cell–cell communication. As cells respond to injury and inflammatory stimuli, targeting part of the ECM is expected, to avoid pathological development and guide wound healing.

## 4. Epigenetic Regulation of ECM in Heart

**Figure 3:**
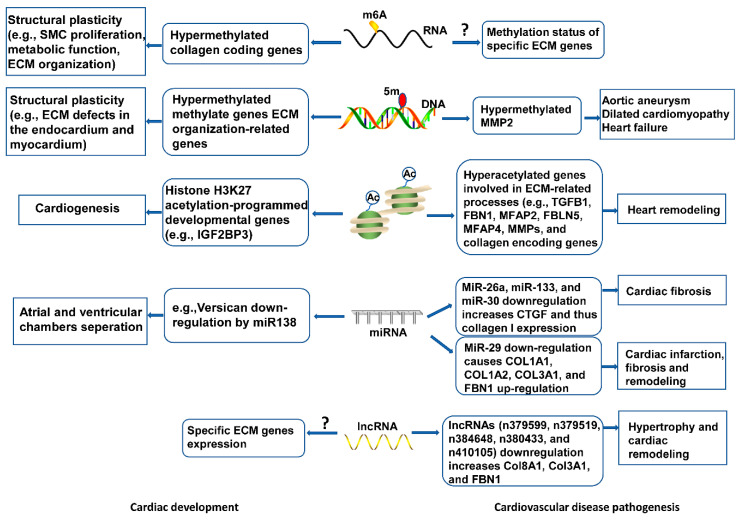
Epigenetic mechanisms of ECM modulation in cardiovascular development and disease. Epigenetic events, including RNA and DNA methylation, histone modifications, and non-coding RNAs mediate ECM gene expression, contributing to cytoskeletal architecture, remodeling, and functional response in heart development. Abnormal epigenetic modification may disrupt ECM homeostasis, leading to cardiovascular pathogenesis.

Emerging research areas in the ECM field include epigenetic control of gene expression of ECM proteins, or indirectly, by modulating the expression of genes that regulate the synthesis or the degradation of ECM molecules in development and disease onset. Epigenetic changes characterized by RNA and DNA methylation, non-coding RNAs-intervened quality guidelines, and histone adjustments, have been seen in cardiovascular dysfunction and heart recovery (Figure 3), yet the components are indistinct. Knowledge of these aspects will deepen our understanding of ECM regulatory roles in cardiac health and disease, and inform new pharmacological agents targeting ECM-related cardiovascular diseases.

### 4.1. RNA and DNA Methylation

RNA modification was first discovered in the 1970s; however, it has newly been considered an epigenetic modification regulating the RNA processing and metabolism related to biogenesis [132,133]. Of which, the m6A modification, which is methylation of the adenosine base at the nitrogen-6 position, was the first identified, and is the most common mRNA methylation in eukaryotes [134,135,136]. Understanding of the role of m6A RNA methylation in cardiovascular development and disease is only emerging [137,138,139]. A recent m6A RNA methylome study revealed m6A RNA methylation changes across hypomethylated and hypermethylated transcripts (e.g., hypermethylated collagen coding genes), and linked to processes of structural plasticity, such as regulation of smooth cell proliferation and metabolic function, as well as ECM organization in human end-stage heart failure [139]. However, how active RNA demethylation is targeted to regulate specific ECM genes during cardiac development and maturation and disease remains largely unknown. Although it is a starting point, it is significant and clinically relevant to further determine m6A’s effects on ECM RNAs in cardiac remodeling-related cardiovascular diseases.

Cardiovascular development and disease are affected by abnormal methylation of CpG islands and medications that repress DNA methyltransferases. Several studies have associated DNA methylation with cardiac development, using both the animal model and in vitro culture systems [140,141]. In a zebrafish model, the tet2/3 mutant failed to demethylate genes associated with ECM organization in the endocardium and myocardium, leading to defects in the ECM for cardiogenesis [142]. Hypermethylated MMP2 was identified and demonstrated to be associated with an increased risk of aortic aneurysm [143]. More recently, in dilated cardiomyopathy, hypomethylations of MMP-2 and connective tissue growth factor (CTGF) were identified as contributing to heart failure [144].

RNA or DNA methylation patterns cause alterations in the ECM gene expression; this may correlate with the increased susceptibility to cardiovascular stress. The reason for this is that they could influence ECM expression and remodeling, which may have an impact on disease development. Such methylation signatures warrant further investigation of RNA and DNA methylation regulated ECM during cardiac health and disease and could be used to discover novel diagnostic and therapeutic targets for cardiovascular disease.

### 4.2. Histone Acetylation

Histone acetylation is linked to unusual phenotypes of heart development, cardiovascular hypertrophy, heart improvement, and contractility. Histone H3K27 acetylation-programmed developmental genes have been identified in both embryonic hearts and the postnatal heart, but not 8-wk-old hearts [145]. Interestingly, overexpression of the developmental gene insulin-like growth factor 2 mRNA binding protein 3 could promote cardiac regeneration, and reduce fibrosis and ventricular dilation in nonregenerative hearts following cardiac MI injury [145]. Together, this provides evidence that epigenetic modification in development may be a potential therapeutic target for cardiovascular disease. Due to the significant role of ECM in heart development, it is likely that histone acetylation of ECM contributes to this dynamic process, and merits being investigated. A recent study of histone acetylome in comparison to remodeled non-failing patient hearts and healthy donor hearts identified essential gene-encoded proteins based on all genes involved in ECM-related processes, including TGFB1, fibrillin-1 (*FBN*)1, microfibril associated protein (MFAP) 2, fibulin 5, MFAP4, a group of MMPs, and a cluster of collagen encoding genes. This study also revealed that the most enriched biological functions in genes close to the hyperacetylated regions were linked to extensive ECM regulation and cell-binding [146]. The role of histone acetylation of ECM in heart remodeling-related cardiovascular disease remains to be further studied.

### 4.3. MicroRNA and Long Non-Coding RNA

MicroRNA (miR) treatments have been proposed for cardiovascular recovery and the multiplication of undifferentiated organisms into cardiomyocytes. miR-17 transgenic mice showed retarded growth rates of the heart, liver, spleen, and whole-body due to the repression of fibronectin and fibronectin type III domain, containing 3A [147]. MiR-138 in the zebrafish heart was demonstrated to suppress versican, a chondroitin sulfate proteoglycan prominent in the heart and vascular system, which contributes to separating atrial and ventricular chambers [148]. MiR-26a, miR-133, and miR-30 have been found to downregulate CTGF expression, contributing to a decrease in expression of collagen type I and suppression of cardiac fibrosis [149,150]. Markedly, in human infarcted cardiac samples, the down-regulation of miR-29 correlated with the up-regulation of collagen genes (*COL1A1, COL1A2, COL3A1*) and *FBN*1 in the infarcted region [151]. Furthermore, an miR-29 mimic down-regulated these collagen mRNA expressions, and suppressed cardiac fibrosis and remodeling [151]. These data support future research in this clinically relevant and promising area.

Long non-coding RNA (lncRNAs) have appeared to take part in pretty much every milestone of cardiovascular breakdown pathogenesis, including ischemic injury, heart hypertrophy, and heart fibrosis. Moreover, the control of lncRNAs whitewashes the movement of cardiovascular breakdown by constricting ischemic heart injury, cardiovascular hypertrophy, and cardiovascular fibrosis, as well as encouraging heart recovery and therapeutic angiogenesis [152]. In left ventricular myocardial samples of patients with ischemic cardiomyopathy, cardiac fibroblast-enriched lncRNAs (n379599, n379519, n384648, n380433, and n410105) were identified. Furthermore, knockdown of these lncRNAs enhanced the expression of COL8A1, COL3A1, and FBN1, which provides evidence that lncRNAs play an essential role in regulating the ECM expression involved in pathological hypertrophy and cardiac remodeling [153].

Together, epigenetic modifications have been shown to play direct and indirect roles in regulating ECM expression, critical mediators in cardiovascular development and remodeling, and implicated in hypertrophy, fibrosis, and heart failure. More studies are needed to elucidate the mechanisms of epigenetic regulation of specific ECM genes, to contribute to developing therapeutic approaches, targeted by reprogramming these modifications.

## 5. Conclusions

The ECM is a crucial element of the heart. Regulation of ECM structural integrity influences the viscus structure and performance. The strict regulation of temporal and spatial expression, and the proteolytic processing of ECM elements by extracellular proteases are crucial for the development of traditional internal organs. ECM pathological transformation is commonly related to viscus pathology alternative adverse outcomes, while the physiological turnover of ECM is beneficial for the process of tissue regeneration and repair.

Imperfect development in the womb is related to the tendency for cardiovascular disease in adulthood, an idea named “developmental origins of health and disease”. More and more evidence supports the association of epigenetic guidelines with the underlying mechanism. Epigenetic systems, for example, RNA and DNA methylation, histone adjustments, and non-coding RNAs, give a degree of quality guidelines without modifying DNA arrangements. These changes are moderately steady signals, offering possible knowledge into the instrument’s fundamental formative starting points of wellbeing and ailment. Therefore, it is imperative to understand the underlying mechanisms of ECM regulation that control cardiovascular development. Understanding the developmental mechanisms of ECM regulation could contribute to developing therapeutic strategies for cardiovascular disease.

## Figures and Tables

**Table 1:** ECM loss-of-function phenotypes in mammalian development.

ECM	Isoform/Type	Receptor	Phenotype	References
Fibronectin		Integrin β1	Early embryonic lethality. Defects in mesodermal, neural tube, and cardiovascular development	[22,23,24]
Laminin	α4	Integrin β1, dystroglycan, and proteoglycans	Defects in microvessel maturation, synaptic maturation	[25,26,27]
	β1	Integrin β1, dystroglycan, and sulfatides	Embryonic lethality. Defects in extraembryonic tissue development, implantation, gastrulation	[28]
	γ1	Integrin β1, dystroglycan, and sulfatides	Embryonic lethality. Defects in endoderm differentiation, axonal sorting and myelination, neurite growth and neuronal migration, extraembryonic tissues development	[29,30,31,32,33]
Collagen	ColI	Integrins, discoidin domain receptors 1 and 2	Embryonic lethality. Defects in circulatory system	[34]
	ColIII		Post-natal death. Defects in cardiovascular system and brain development	[35,36]
	ColIV		Embryonic lethality. Defects in basement membrane integrity and capillary structures and renal development	[37,38]
	ColV		Early embryonic lethality. Defects in fibril formation, and ventricular myocardial morphogenesis and heart valve development	[39,40,41]
	ColXI		Defects in skeletal morphogenesis, and ventricular myocardial morphogenesis and heart valve development	[41,42]
	ColXIV		Defects in fiber and fibril assembly in tendons, and growth and structural integrity of the myocardium	[43,44]
	ColXV		Defects in skeletal muscle and cardiovascular development, and axonal segregation and myelination	[45,46]
Elastin		Galectin-3, integrins, and elastin receptor complex comprising the elastin binding protein, the protective protein/cathepsin A and the membrane-bound neuramidase-1	Post-natal death. Defects in cardiovascular morphogenesis and development	[47,48,49]
Fibrillin	FBN1	Integrins	Post-natal death. Defects in cardiovascular development and integrated tendon formation	[50,51]
Fibulin	Fibulin-1	Integrins	Perinatal lethal. Defects in vascular, lung and kidney development	[52,53]
	Fibulin-4		Defects in elastogenesis in lungs and vasculature, and cardiovascular development	[54,55,56]
	Fibulin-5		Defects in elastogenesis in the skin, lung and vasculature	[57,58]
Tenascin	TNC	Integrins	Defects in neural development, alveolarization and microvascular maturation	[59,60,61]
Versican		CD44, integrins, epidermal growth factor receptor, and P-selectin glycoprotein ligand-1	Embryonic lethality. Defects in heart and neural development	[62,63,64]
Thrombospondin	TSP-4	Integrins	Increased production of ECM and enlarged heart	[65]

## Abbreviations

CAR	Chimeric antigen receptor
CCL2	C-C motif chemokine ligand 2
COVID-19	Coronavirus disease 2019
CTGF	Connective tissue growth factor
CXCL5	C-X-C motif chemokine ligand 5
ECM	Extracellular matrix
FAP	Fibroblast activation protein
FBN	Fibrillin-1
FN	Fibronectin
HA	Hyaluronic acid
Hapln1	Hyaluronan and proteoglycan link protein 1
LncRNA	Long non-coding RNA
LV	Left ventricular
MFAP	Microfibril associated protein
MI	Myocardial infarction
MiR	MicroRNA
MMPs	Matrix metalloproteinases
PGP	Pro-Gly-Pro
Postn	Periostin
TNC	Tenascin C
TSP	Thrombospodin
